# Association of Soluble Suppression of Tumorigenesis-2 (ST2) with Endothelial Function in Patients with Ischemic Heart Failure

**DOI:** 10.3390/ijms21249385

**Published:** 2020-12-09

**Authors:** Stathis Dimitropoulos, Vasiliki Chara Mystakidi, Evangelos Oikonomou, Gerasimos Siasos, Vasiliki Tsigkou, Dimitris Athanasiou, Nikolaos Gouliopoulos, Evanthia Bletsa, Aimilios Kalampogias, Georgios Charalambous, Costas Tsioufis, Manolis Vavuranakis, Dimitris Tousoulis

**Affiliations:** 1 First Department of Cardiology, ‘Hippokration’ General Hospital, School of Medicine, National and Kapodistrian University of Athens, 11528 Athens, Greece; stakyp@yahoo.com (S.D.); xaram25@gmail.com (V.C.M.); ger_sias@hotmail.com (G.S.); bikytsigkoy@yahoo.gr (V.T.); dimitris.eathanasiou@yahoo.gr (D.A.); ngouliopoulos@yahoo.gr (N.G.); evabletsa@gmail.com (E.B.); akalamp@gmail.com (A.K.); drcharalambous@yahoo.gr (G.C.); tsioufis@hippocratio.gr (C.T.); drtousoulis@hotmail.com (D.T.); 2 Third Department of Cardiology, Medical School, National and Kapodistrian University of Athens, 15772 Athens, Greece; vavouran@otenet.gr

**Keywords:** heart failure, soluble suppression of tumorigenesis-2, endothelial function, FMD

## Abstract

Soluble suppression of tumorigenesis-2 (sST2) has been introduced as a marker associated with heart failure (HF) pathophysiology and status. Endothelial dysfunction is a component underlying HF pathophysiology. Therefore, we examined the association of arterial wall properties with sST2 levels in patients with HF of ischemic etiology. We enrolled 143 patients with stable HF of ischemic etiology and reduced left ventricular ejection fraction (LVEF) and 77 control subjects. Flow-mediated dilation (FMD) was used to evaluate endothelial function and pulse wave velocity (PWV) to assess arterial stiffness. Although there was no significant difference in baseline demographic characteristics, levels of sST2 were increased in HF compared to the control (15.8 (11.0, 21.8) ng/mL vs. 12.5 (10.4, 16.3) ng/mL; *p* < 0.001). In the HF group, there was a positive correlation of sST2 levels with age (rho = 0.22; *p* = 0.007) while there was no association of LVEF with sST2 (rho = −0.119; *p* = 0.17) nor with PWV (rho = 0.1; *p* = 0.23). Interestingly, sST2 was increased in NYHA III [20.0 (12.3, 25.7) ng/mL] compared to patients with NYHA II (15.0 (10.4, 18.2) ng/mL; *p* = 0.003) and inversely associated with FMD (rho = −0.44; *p* < 0.001) even after adjustment for possible confounders. In patients with chronic HF of ischemic etiology, sST2 levels are increased and are associated with functional capacity. There is an inverse association between FMD and sST2 levels, highlighting the interplay between the dysfunctional endothelium and HF pathophysiologic mechanisms.

## 1. Introduction

The prevalence of heart failure (HF) worldwide is approximately 1% to 2% and it is estimated that it exceeds 10% in subjects over 70 years old [1,2]. Natriuretic peptides—b-type natriuretic peptide (BNP) and N terminal pro BNP (NTproBNP)—and cardiac troponins (Tn) I and T are found to be elevated in HF following increased myocardial wall stress, elevated filling pressures, subendocardial ischemia etc. [3,4]. Moreover, they are associated with prognosis and disease severity [4,5,6].

The European Society of Cardiology has already announced research on novel HF biomarkers so as to be used in clinical practice, as a multimarker approach is preferred over the old fashion single biomarker approach [7]. New biomarkers are those one of inflammation, oxidative stress, vascular dysfunction, and myocardial remodeling. They have been proposed as indices related to HF status, functional capacity, and prognosis [4,8,9]. Galectin-3 (Gal–3), soluble suppression of tumorigenesis-2 (sST2), and high-sensitivity cardiac troponin (hs–cTn) are mainly predictors of hospitalization and death in HF patients, and in addition to NPs can increase the prognostic value [10,11]. To this direction, sST2 has been introduced as a marker associated with acute decompensate heart failure, pro-inflammatory status, endothelial dysfunction, myocardial fibrosis. and adverse remodeling with prognostic capability [12]. Additionally, sST2 has a low biological variability and a low index of individuality (0.25), favorable characteristics that may be used for guiding therapy and monitoring HF patients [13,14,15,16,17].

Endothelial dysfunction is considered an initial step in the process of atherosclerosis and coronary artery disease progression and is considered as a component underlying HF pathophysiology. Endothelial dysfunction plays an important role in HF progression. It worsens the vasoconstriction and increases myocardial damage. Dysfunctional endothelium increases the afterload due to systemic and pulmonary vascular constriction. Myocardial perfusion is also impaired due to decreased coronary endothelium-dependent vasodilation [8,9,18].

Since ST2 is produced among other cells by endothelial cells of cardiac vasculature [19], in this study, we examined the association of arterial wall properties and endothelial function with sST2 levels in patients with HF of ischemic etiology.

## 2. Results

### 2.1. Demographic and Clinical Characteristics

As it is shown in Table 1, the mean age of the HF patients was 67 ± 12 years. From the HF patients 68% were categorized as NYHA class II. The median ejection fraction was 30% (25%, 40%). More than half of them (56%) had hypertension and 52% diabetes mellitus (DM).

In the control group, the mean age was 63 ± 10 years. As far as their medical history is concerned, 32% had hypertension, 37% had DM, and 20% had hyperlipidemia.

Between the two groups, there was no significant difference in age. The prevalence of DM (52% vs. 37%; *p* = 0.02) and hypertension (56% vs. 32%; *p* = 0.001) was higher in HF subjects compared to the control while there was no significant difference regarding the history of hyperlipidemia.

Significant differences between HF and control subjects were observed regarding treatment. The majority of HF subjects were under diuretics, angiotensin converting enzyme inhibitors (ACEIs), or angiotensin II receptor blockers (ARBs), and β–blockers.

**Figure 1:**
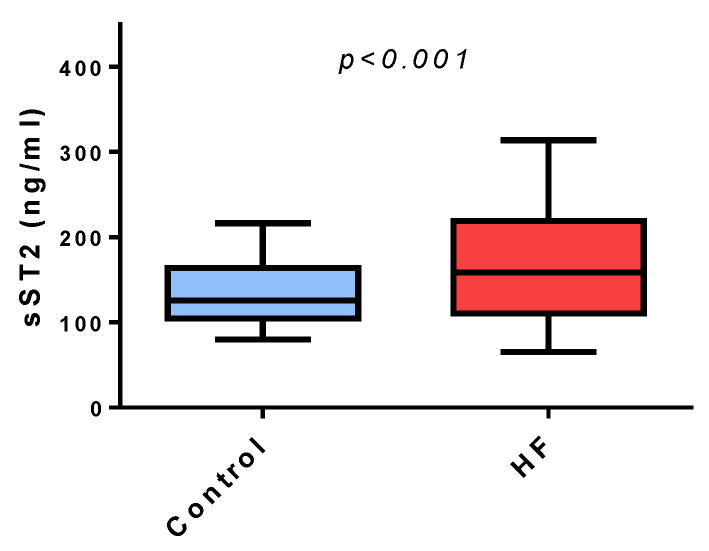
Serum levels of soluble Suppression of Tumorigenesis-2 are higher in the heart failure group compared to the control group. The Distribution of serum levels of soluble Suppression of Tumorigenesis-2 are shown with Box-plots sST2: Soluble Suppression of Tumorigenesis-2.

Serum levels of urea and creatine was higher in subjects with HF compared to control. Serum ICAM-1 levels was higher in the HF group compared to the control group (276 (221, 315 ng/mL vs. 212 (175, 267) ng/mL, *p* < 0.001). NT pro-BNP in the HF group (140 (96, 290) pg/mL) was increased compared to the control group (50 (32, 82) pg/mL; *p* < 0.001). sST2 was higher in the HF group (15.8 (11.0, 21.8) ng/mL) compared to the control group (12.5 (10.4, 16.3) ng/mL; *p* < 0.001) (Figure 1).

### 2.2. Factors Affecting sST2 Level in Subjects with Heart Failure

To examine how sST2 levels may be affected by various demographic, clinical, and laboratory factors, we examined in a univariate fashion the correlation of sST2 with multiple factors in the HF population.

### 2.3. Association of sST2 with Demographic Characteristics of Heart Failure Subjects

In subjects with HF, there was a positive correlation of sST2 with age (rho = 0.22; *p* = 0.007) but not with body mass index (rho = −0.094; *p* = 0.42). The sST2 levels did not differ between female and male subjects (15.4 (11.1, 18.7) ng/mL vs. 14.2 (10.3, 19.9) ng/mL; *p* = 0.72), between hypertensive and normotensive subjects (15.2 (10.9, 21.70 ng/mL vs. 12.9 (10.2, 17.4) ng/mL; *p* = 0.08), and between subjects with DM compared with normo-glycemic subjects (14.5 (10.6, 20.1) ng/mL vs. 13.5 (10.5, 18.7) ng/mL; *p* = 0.45) (Supplementary Figure S1A–E).

### 2.4. Association of sST2 with Clinical Characteristics in Subjects with Heart Failure

In subjects with HF, sST2 levels were not associated with LVEF (rho = −0.119; *p* = 0.17). Regarding the NYHA functional classification in the HF group, sST2 was higher in NYHA III (20.0 (12.3, 25.7) ng/mL) compared to patients with NYHA II (15.0 (10.4, 18.2) ng/mL; *p* = 0.003). The sST2 levels were not affected by the use or not of ACEI or ARB (15.1 (11.2, 22.9) ng/mL vs. 16.0 (7.8, 20.7) ng/mL; *p* = 0.46), by the use or not of mineralocorticoid receptor antagonist (MRA) (16.0 (9.9, 20.6) ng/mL vs. 12.9 (10.3, 18.5) ng/mL; *p* = 0.77), and by the use or not of β–blockers (14.8 (9.5, 21.8) ng/mL vs. 17.4 (11.2, 22.3) ng/mL; *p* = 0.63). sST2 levels were inversely associated with eGFR (rho = −1.75; *p* = 0.03). sST2 was not associated with CRP (rho = 0.45; *p* = 0.26), TNFa (rho = −0.024; *p* = 0.77), ICAM–1 (rho = 0.02, *p* = 0.84), or NTproBNP (rho = 0.92; *p* = 0.27) (Supplementary Figure S1F–M).

**Figure 2:**
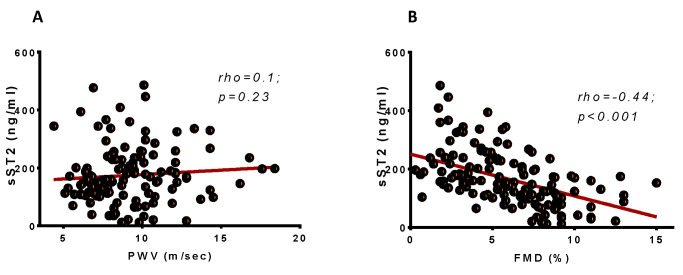
Levels of soluble suppression of Tumorigenesis-2 are inversely associated with FMD but not with PWV. (**A**) Scatter-dot of the association of PWV with soluble suppression of Tumorigenesis-2. (**B**) Scatter-dot of the association of FMD with soluble suppression of Tumorigenesis-2. sST2: Soluble Suppression of Tumorigenesis-2; PWV: Pulse wave velocity; FMD: Flow-mediated dilation.

sST2 was not associated with arterial stiffness—PWV (rho = 0.1; *p* = 0.23) (Figure 2A). Interestingly, sST2 was inversely associated with FMD (rho = −0.44; *p* < 0.001) (Figure 2B). To further test how sST2 levels are affected by endothelial function (FMD), independently from other confounders, we proceeded to a linear regression analysis in which we included all variables proved significant in the univariate analysis (Table 2). sST2 was, independently of other confounders, inversely associated with FMD in patients with HF of ischemic etiology, and for every increase in FMD by 1%, there is an anticipated decrease in sST2 levels by approximately 14 ng/mL.

## 3. Discussion

In the present study, we examined the association between biomarkers of cardiovascular stress and interstitial fibrosis with vascular function in stable patients with systolic HF of ischemic etiology. We found that sST2, a novel HF biomarker associated with myocardial fibrosis, produced by either myocardial cells, fibroblasts or, endothelial cells, and prognosis [20,21,22] is correlated with FMD, which expresses the status and health of the endothelial cells layer, highlighting the key role of the endothelium in the progress of HF [8,9].

### 3.1. sST2 in Heart Failure

sST2 has been initially identified as a marker of myocyte stress [23]. sST2 assays are accurate with high repeatability [24], implying the possible use of sST2 as an additional clinical meaningful biomarker.

We identified a significant difference on sST2 levels between control and HF subjects, although there was significant overlap in the values observed in the two examined groups, which is in accordance with previous analytical studies on sST2 and the Framingham Heart Study [24,25]. Therefore, sST2 cannot be used in the etiologic diagnosis of dyspnea.

Beyond the role of sST2 as a surrogate of myocardial stress, sST2 is mainly produced by extracardiac tissues (i.e., alveolar cells, fibroblasts, vessel wall cells) [26,27,28]. As a response to the continuous stress stimulus, there is an upregulation in ST2 gene expression. Concerning sST2 release from alveolar epithelial cells, there is an association between the increase in alveolus thickness and the upregulation of sST2, which suggests a relationship between the severity of pulmonary edema congestion and alveolar strain with sST2 production [26]. Inflammatory and pro-fibrotic stimulus are also considered responsible for the activation of ST2 production and release in the circulation [26,27,28]. sST2 is a circulating receptor for interleukin 33 (Il-33). The connection between Il-33 and ST2 ligand provokes anti-inflammation and antithrombotic processes in the damaged heart. Therefore, sST2 is a decoy receptor of Il-33 and attenuates the beneficial effects of Il-33 connection to ST2 ligand, boosting inflammatory and thrombotic damage to the heart [29].

In our HF study population, we found that sST2 levels were associated with HF status and functional capacity of the patients as assessed with NYHA classification. Indeed, patients with functional impairment have been identified with higher values of sST2, although the levels of circulating sST2 were not associated with LVEF. These findings may be explained since in chronic HF, sST2 levels are mainly associated with LV diastolic dysfunction and increased left and right ventricular pressures, which are key determinants of the functional capacity of patients [30]. However, in our cohort of chronic HF patients, we did not identify any association of sST2 with NTproBNP levels. The lack of association can be attributed to the specific characteristics of our study population especially regarding the stable clinical condition for at least 3 months. Indeed, we excluded from the study subjects with recent decompensation or subjects not on optimal medical treatment and therefore with NT-pro-BNP levels at the lower rate.

Impaired renal function is a significant determinant of HF prognosis and is associated with myocardial fibrosis [31]. sST2 is associated with adverse prognosis in subjects with chronic kidney disease [31,32]. We found that in the HF population, sST2 was inversely associated with estimated GFR, implicating the cardiorenal axis and the bidirectional toxic effects of volume overload in the heart and kidneys.

### 3.2. sST2 and Vascular Wall Properties

Arterial wall properties constitute the second component of the cardiovascular system and have a key role in the cardiovascular homeostasis. They regulate vasomotor activity, arterial stiffness, afterload, and consequently cardiac function [33]. Impaired endothelial function has been proposed as a factor implicated in the development and progress of HF especially of ischemic etiology and several approaches may beneficially affect the endothelium in the concept of HF [34,35,36]. Systemic vasoconstrictor is observed in chronic HF settings and decreased endothelium-dependent vasodilatation may be the underlying pathophysiologic background contributing to a lower cardiac output state.

We found that in HF patients, there is an inverse association between endothelial function and sST2 levels. sST2 is produced among other cells by endothelial cells and alveolar epithelium [37]. The inverse association found in our study may be confirmatory that dysfunctional endothelium (under the stimulus of decrease shear stress, proinflammatory milieu, and oxidative stress) may lead to highly expressed levels of sST2, which in turn may have detrimental effects on cardiac function and remodeling. Indeed, even after adjustment for potential confounders associated with functional capacity, the health of the endothelial layer was a significant contributor of sST2 levels.

As opposed to endothelial function, we did not observe any association of sST2 levels with PWV and arterial stiffness a surrogate of arteriosclerosis in subjects with HF of ischemic etiology [38,39]. Although sST2 levels in patients with coronary artery disease have been associated with aortic stiffness, in our study population with stable chronic HF, we only identified a link between endothelial function and sST2 levels. In the same direction, we did not find an association between serum ICAM-1 levels and sST2 in our populations of HF subjects despite the well-described effects of adhesion molecules in endothelial dysfunction [40]. In HF, beyond the role of leukocytes and inflammatory milieu, underlying mechanisms may contribute to impairment of endothelium (i.e., low arterial shear stress, oxidative stress) [8].

### 3.3. Clinical Importance

Although sST2 levels cannot be used for diagnosis of HF, they are significantly associated with prognosis and functional capacity [41,42]. The association of endothelial function with sST2 levels emphasizes the role of the vascular system as an important determinant of functional capacity beyond left ventricle systolic performance. It may also imply a link between dysfunctional endothelium in the pulmonary circulation associated with congestion in patients with HF and emphasizes the systemic nature of HF syndrome, suggesting that treatment of underlying pathologic conditions may affect HF course.

### 3.4. Limitations

Although, in our study, we achieved a match case control population, the design of our study contains inherent limitations. Accordingly, we cannot conclude on the importance of ST2 on prognosis or diagnosis of HF. Moreover, based on our study findings, we cannot conclude on a straightforward association of ST2 levels with endothelial function and we cannot provide etiologic or pathophysiologic insights on the mechanisms underlying the link between ST2 and endothelial dysfunction in subjects with HF.

## 4. Methods

### 4.1. Study Population

In the period from June 2018 to March 2019, 143 patients with stable HF of ischemic etiology and reduced left ventricular ejection fraction (LVEF), as assessed by echocardiogram, were enrolled for the purpose of this study.

A group of 77 control subjects were also recruited from the outpatient cardiology department, where they were referred for preventive examination. All the recruited control patients had no symptoms or signs of heart failure and normal ejection fraction, had a normal physical examination, normal electrocardiogram, and a normal LVEF (>55%).

### 4.2. Data Collection and Biochemical Measurements

Demographics were collected in all subjects by the use of standard questionnaires and procedures. Clinical characteristics, and data regarding ECG, echocardiography, coronary angiography (in patients with HF of ischemic etiology), biochemistry, NTproBNP, high-sensitivity C–reactive protein (hsCRP), and pharmacotherapy were also collected.

Standard transthoracic echocardiographic examination was carried out in all subjects by the same expert using a vivid e-cardiovascular ultrasound system (General Electric, Milwaukee, WI, USA) equipped with a 2.0–3.6 MHz (harmonics) phased array transducer. Left ventricle ejection fraction was calculated by biplane Simpson’s modified rule as previously described. All measurements were performed according to the recommendations of the American Society of Echocardiography and the European Association of Cardiovascular Imaging. The diagnosis of coronary artery disease was established by a history of myocardial infarction or by evidence of a coronary vessel luminal stenosis >75% as detected by coronography, either done in a previous hospitalization or in hospitalization during the enrollment of the patient in the study. Subjects finally enrolled in the study were under optimal medical treatment and under stable clinical status for at least three months prior to their entry in the study, as characterized by NYHA classification.

A fasting venous blood sample was taken from each individual by venipuncture between 8.00 and 10.00 a.m. Samples were centrifuged at 3000 rpm and serum/plasma was collected and stored at −80 °C until assayed. sST2 measured with a Presage^ΤΜ^ ST2 Assay (Critical Diagnostics, San Diego, CA, US). Calibration and standardization of these assays were performed according to the manufacturers’ protocols. NTproBNP concentrations were measured quantitatively using a fluorescence immunoassay with a single-use device (Triage BNP Test; Biosite, Inc., San Diego, CA, USA). Tumor necrosis factor alpha (TNF-α) and intracellular adhesion molecule-1 (ICAM-1) levels in the serum were measured with enzyme link immunosorbent assays (ELISA). The Modification of Diet in Renal Disease study (MDRD) formula was used to estimate the glomerular filtration rate (eGFR).

Endothelial function was evaluated by estimating the flow-mediated dilation in the brachial artery [42]. In brief, after a 10-min rest, the right brachial artery was scanned in the longitudinal section, 5 cm above the antecubital fossa, using a Vivid e ultrasound system (General Electric, Milwaukee, WI, USA) equipped with a 5.0–13.0 MHz (harmonics) linear array ultrasound transducer. A pneumatic cuff placed distal to the ultrasound probe was then inflated to suprasystolic pressure on the forearm for 5 min to induce reactive hyperemia. After the release of the ischemia cuff, brachial artery diameter was measured manually with electronic calipers (as the average derived from multiple diameter measurements along a segment of the vessel) at the boundaries of the media–adventitia interfaces, every 15 s for 2 min, and FMD was defined as the % change of vessel diameter from rest to the maximum diameter following cuff release. The same examiner throughout the study conducted examinations. The same observer who was blinded to the image sequence assignment proceeded to all measurements of brachial artery diameter. Endothelium-independent dilation (EID) was defined as the % change of vessel diameter from rest to the maximum diameter post sublingual nitrate given.

Arterial stiffness was evaluated in all patients with pulse wave velocity (PWV) measurements. Carotid-femoral pulse wave velocity (PWV), which is considered to be an index of aortic stiffness, was calculated from measurements of the pulse transit time and the distance traveled between 2 recording sites (PWV = distance in meters divided by transit time in seconds) by using a well-validated noninvasive device (SphygmoCor; AtCor Medical, Sydney, NSW, Australia). Two different pulse waves were obtained at 2 sites (at the base of the neck for the common carotid and over the right femoral artery) with the transducer. Distance was defined as the distance from the suprasternal notch to the femoral artery minus the distance from the carotid artery to the suprasternal notch [38,42].

### 4.3. Bioethics

All subjects were informed about the aims of the study and gave their written informed consent. The study was approved by the local ethics committee of our institution (15 July 2014) and was carried out in accordance with the Declaration of Helsinki (1989).

### 4.4. Statistical Analysis

All variables were tested for normal distribution of the data using the P–P plots and Shapiro–Wilk test. Data are expressed as means ± standard deviation, when normally distributed otherwise, as median with interquartile range. Data not normally distributed were logarithmically transformed to improve normality and log-transformed data were used if normality was achieved. Differences between groups of subjects were tested with a t-test and chi square test for continuous and categorical variables, respectively. Spearman correlation was used to test for an association between continuous variables. A linear regression model was applied to test the association of FMD on sST2 independently from other established confounders (i.e., age, sex) or variables proved significant in the univariate analysis. Statistical analysis was performed using SPSS version 25 (IBM SPSS Statistics Version 25.0. Armonk, NY, USA).

## 5. Conclusion

In patients with chronic HF of ischemic etiology, sST2 levels are increased and are associated with functional capacity and renal impairment. There is an inverse association between FMD and sST2 levels, highlighting the interplay between dysfunctional endothelium interstitial fibrosis and the HF pathophysiologic mechanism as they can be evaluated by circulating sST2 levels.

## Figures and Tables

**Table 1:** Study participants’ demographic, clinical, and laboratory characteristics.

−	HF Group	Control Group	*p*-Value
Age (years)	67 ± 12	68 ± 10	0.45
Male gender (%)	85	81	0.439
BMI (kg/m^2^)	28.2 ± 6.5	27.1 ± 3.5	0.055
DM (%)	52	37	0.02
Hypertension (%)	56	32	0.001
Hyperlipidemia (%)	60	20	0.08
NYHA 2 (%)	68	−	−
ACEI or ARB (%)	66	17	0.017
MRA (%)	58	3	<0.001
Diuretics (%)	77	13	<0.001
Β-blockers (%)	88	29	<0.001
eGFR (mL/min)	78.5 (54.3, 108.8)	87.8 (71.1, 114.7)	0.48
Serum Urea (mg/dl)	44 (30, 69)	27 (24, 31)	<0.001
Serum Creatinine (mg/dl)	1.0 (0.9, 1.3)	0.8 (0.7, 1.0)	<0.001
NTproBNP (pg/mL)	140 (96, 290)	50 (32, 82)	0.001
TNFa (ng/mL)	2.4 (1.4, 2.7)	0.7 (0.6, 0.8)	<0.001
ICAM-1 (ng/mL)	276 (221, 315)	212 (175, 267)	<0.001
PWV (m/s)	8.7 (7.2, 10.6)	8.2 (7.4, 9.0)	0.01
FMD (%)	5.6 (3.2, 8.0)	6.1 (3.7, 8.3)	0.71
EF (%)	30 (25, 40)	55 (55, 60)	<0.001
sST2 (ng/mL)	15.8 (11.0, 21.8)	12.5 (10.4, 16.3)	<0.001

HF: Heart failure; BMI: Body Mass Index, DM: Diabetes Meletus, NYHA: New York Heart Association Classification, ACEI: Angiotensin Converting Enzyme Inhibitors, ARB: Angiotensin II Receptor Blockers, MRA: Mineralocorticoid Receptor Antagonists, eGFR: estimated Glomerular Filtration Rate, NTproBNP: N terminal pro hormone B-type Natriuretic Peptide, EF: Ejection Fraction, sST2: soluble Suppression of Tumorigenesis-2, TNFa: Tumor Necrosis Factor a, PWV: Pulse Wave Velocity, FMD: Flow-mediated Dilation; ICAM-1: Intracellular adhesion molecule 1.

**Table 2:** Multiple linear regression analysis for the association of sST2 (dependent variable) with several variable.

Variables	B Coefficient	95% CI	*p*-Value
Age (years)	1.35	−0.87, 3.55	0.22
Sex	29.91	−13.85, 73.68	0.17
eGFR	0.11	−0.56, 0.80	0.73
NYHA class	−	−	−
NYHA II	−	−	−
NYHA III	15.76	−2.09, 33.62	0.08
FMD (%)	−14.12	−20.02, −8.22	<0.001

eGFR: estimated Glomerular Filtration Rate, NYHA: New York Heart Association Classification, FMD: Flow-mediated dilation. For Sex, the reference category was set as female; For NYHA, the reference category was the NYHA 2 stage.

## Supplementary Materials

Supplementary materials can be found at https://www.mdpi.com/1422-0067/21/24/9385/s1, Figure S1: Multi-panel figure (A–K) showing how sST2 levels are associated with continuous and categorical variables in the heart failure group.

## Abbreviations

sST2	Soluble Suppression of Tumorigenesis-2
HF	Heart failure
LVEF	Left ventricular ejection fraction
FMD	Flow mediated dilation
PWV	pulse wave velocity
BNP	b–type natriuretic peptide
NTproBNP	N terminal pro BNP
Tn	cardiac troponins
DM	diabetes mellitus
ACEI	Angiotensin Converting Enzyme Inhibitors
ARB	Angiotensin II Receptor Blockers
ECG	electrocardiogram
hsCRP	sensitivity C–reactive protein
BMI	Body Mass Index
NYHA	New York Heart Association Classification
MRA	Mineralocorticoid Receptor Antagonists
eGFR	estimated Glomerular Filtration Rate
EF	Ejection Fraction
TNFa	Tumor Necrosis Factor a
ICAM-1	Intracellular adhesion molecule 1
