# Supplementary material for: Association of Soluble Suppression of Tumorigenesis-2 (ST2) with Endothelial Function in Patients with Ischemic Heart Failure
Source: Int J Mol Sci. 2020 Dec 9;21(24):9385. doi: 10.3390/ijms21249385 (PMC7764062; doi:10.3390/ijms21249385)
Supplement: Supplementary file 1 [file ijms-21-09385-s001.pdf]

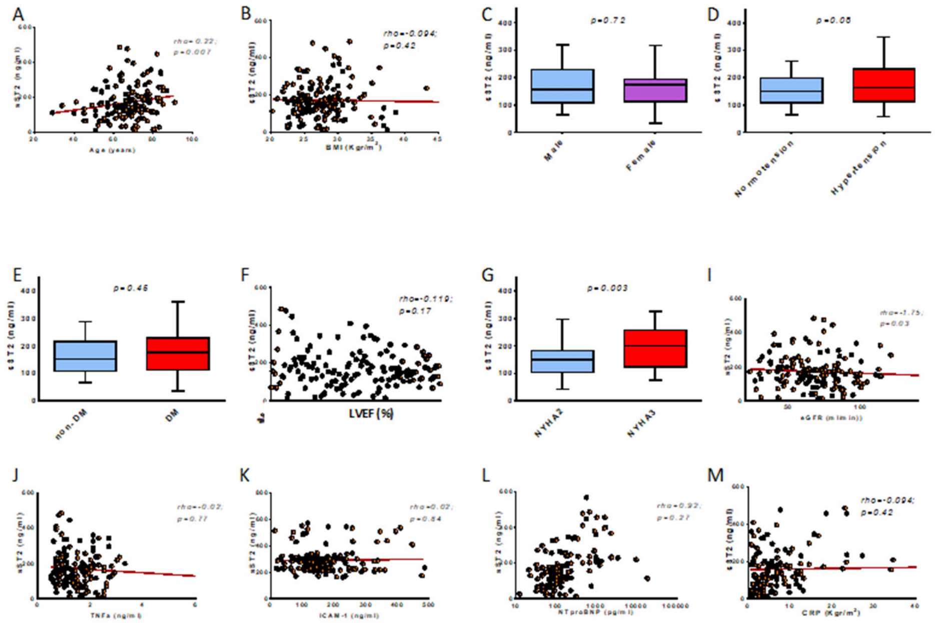

Supplementary Figure 1: Multi-panel figure (**A**–**K**) showing how sST2 levels are associated with continuous and categorical variables in the heart failure group. Box-plots are used for categorical variables and scatter-dots graphs for continuous variables. sST2: Soluble Suppression of Tumorigenesis-2; BMI: Body mass index; DM: Diabetes mellitus; LVEF: Left ventricle ejection fraction; NYHA: New York Heart Association; eGFR: estimated Glomerular Filtration rate; TNFα: Tumor necrosis factor alpha; NTproBNP: N terminal pro B type natriuretic peptidel CRP: C ractive protein.
